# Out of Sight, Not Out of Mind: Vulval Fibroadenoma Revealed

**DOI:** 10.7759/cureus.76571

**Published:** 2024-12-29

**Authors:** Ketan Banerjee, Megha Tandon, Amit Yadav, Mayank Kushwaha

**Affiliations:** 1 Department of Surgery, Vardhman Mahavir Medical College (VMMC) & Safdarjung Hospital, New Delhi, IND; 2 Department of Pathology, Vardhman Mahavir Medical College (VMMC) & Safdarjung Hospital, New Delhi, IND

**Keywords:** case report, ectopic breast tissue, fibroadenoma, rare breast anomalies, vulval fibroadenoma

## Abstract

Ectopic breast tissue (EBT) represents a congenital anomaly caused by incomplete regression of mammary ridges at the time of embryonic development. Typically, EBT presents along the mammary line, although usually in the axillary region, it has been located occasionally in unusual sites such as the vulva. Though relatively rare, it is generally subject to a wide range of pathologies that afflict normal breast tissue, encompassing both benign and malignant transformations. Vulval fibroadenoma is a benign tumor originating from EBT and represents a particularly rare manifestation, with only a few cases reported in the medical literature.

This report presents a case of a 35-year-old female patient presenting with cyclical pain in both breasts and a painful lump in the left vulvar region that had grown insidiously over a period of one year. Following the principles of triple assessment and physical examination and investigations for the vulval lump, it was diagnosed to be a fibroadenoma within ectopic breast tissue in the vulva.

This case highlights the diagnostic challenge presented by this rare site of EBT in the vulva and has pointed out that EBT should be considered as a cause of vulvar mass. Because of the malignant change that may occur in EBT, early diagnosis and treatment are of paramount importance. This report adds to the few documented cases of EBT of the vulva and increases awareness among clinicians so as not to miss or delay such a rare condition.

## Introduction

Ectopic breast tissue (EBT), a congenital anomaly, refers to mammary tissue found outside the normal breast [1]. It originates from incomplete regression of mammary ridges during gestation, presenting along the milk line, notably in the axilla [2]. Clinical manifestations vary, from asymptomatic masses to fully functional breasts, sometimes leading to pain and swelling [3]. While primarily benign, EBT carries a rare risk of malignancy, necessitating timely diagnosis and management.

Diagnostic evaluation involves clinical examination and imaging modalities such as ultrasound (USG) and MRI, often followed by histopathological analysis. Treatment strategies range from observation to surgical excision, depending on symptoms and patient preference [2]. Despite its rarity, EBT poses diagnostic and therapeutic challenges, emphasizing the need for a comprehensive understanding [3]. In this report, we present a case of EBT in a 35-year-old woman, discussing diagnostic and therapeutic approaches, alongside a pertinent literature review [4].

## Case presentation

**Figure 1:**
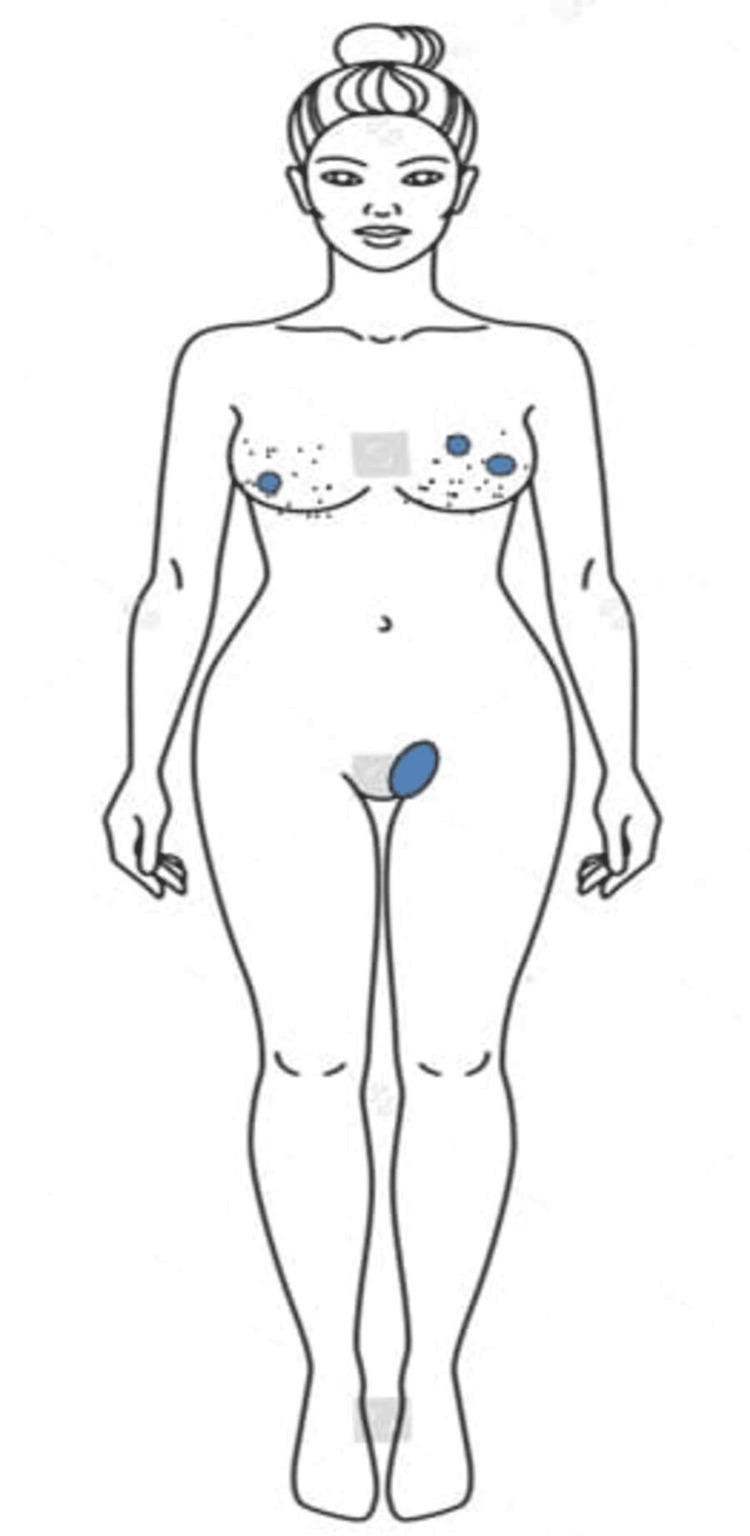
Location of the breast and vulval lumps (indicated in blue colour) Original image created by the authors using Microsoft PowerPoint (Microsoft Corp., Redmond, WA).

A 35-year-old lady presented to the surgery outpatient clinic with complaints of bilateral cyclical mastalgia and a lump in the left vulvar area for the past year. The vulvar lump was painful and gradually increased in size over the last year to reach a size of 3x2 cm at presentation. The patient had no comorbidities, menstrual irregularities, or history of hormonal treatment or contraceptive use. There was no history of alcohol or tobacco consumption either. On physical examination of the breasts, both were nodular with well-defined, firm, smooth, mobile, and slightly tender swellings noted at the seven o'clock position in the right breast and three and 11 o'clock position in the left breast (Figure 1). No skin changes over the breasts were seen. The left vulvar swelling was 3x2 cm in size, ovoid in shape, with a smooth surface with well-defined margins, firm in consistency, non-tender, and mobile. The swelling was not compressible, had no visible or palpable cough impulse, was not fixed to the overlying skin, nor did it have any visible punctum or scars on the skin above the swelling (Figure 1).

**Figure 2:**
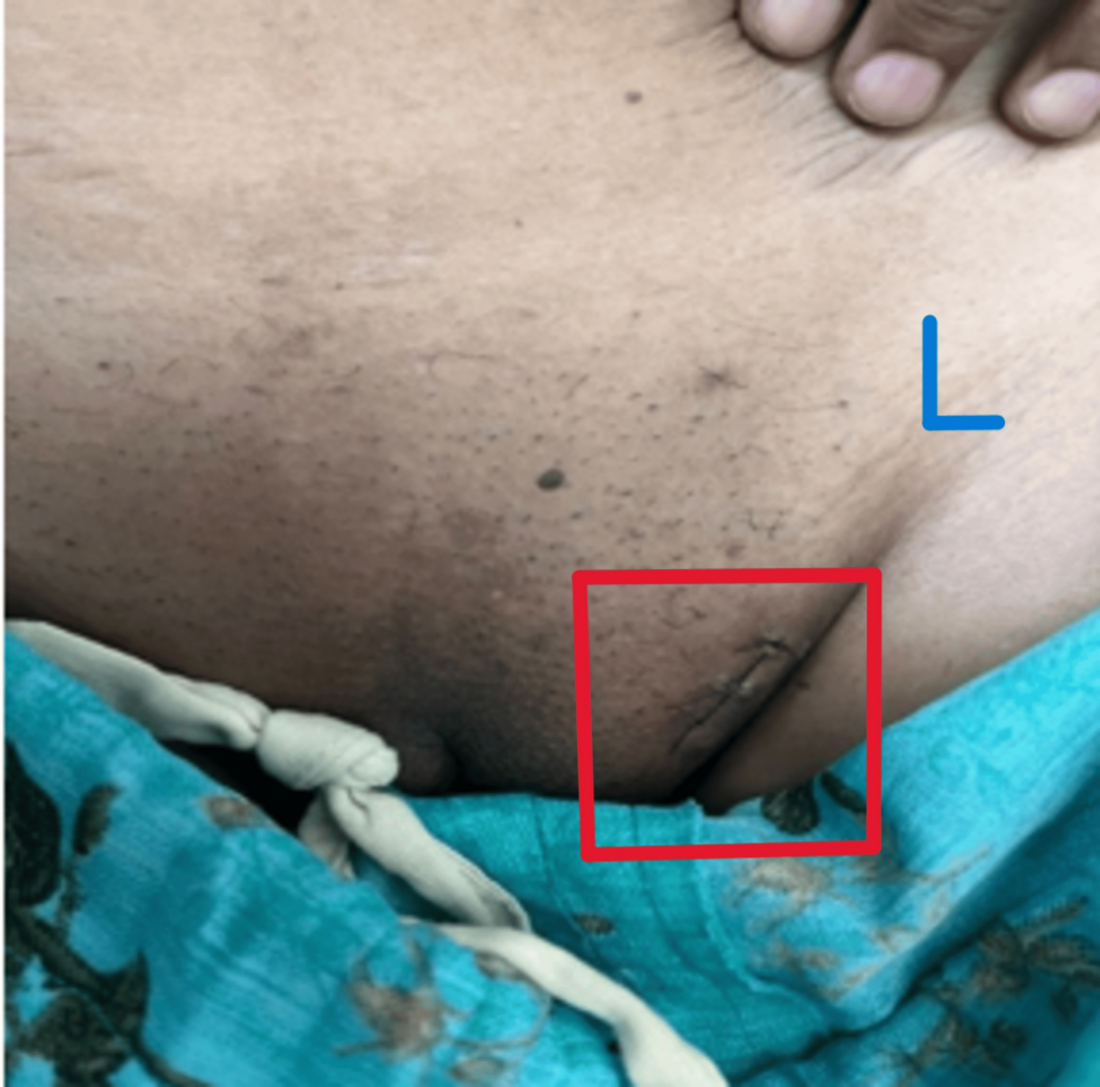
Image obtained after the excision of the vulval fibroadenoma L: left side; the red box indicates the postoperative wound.

**Figure 3:**
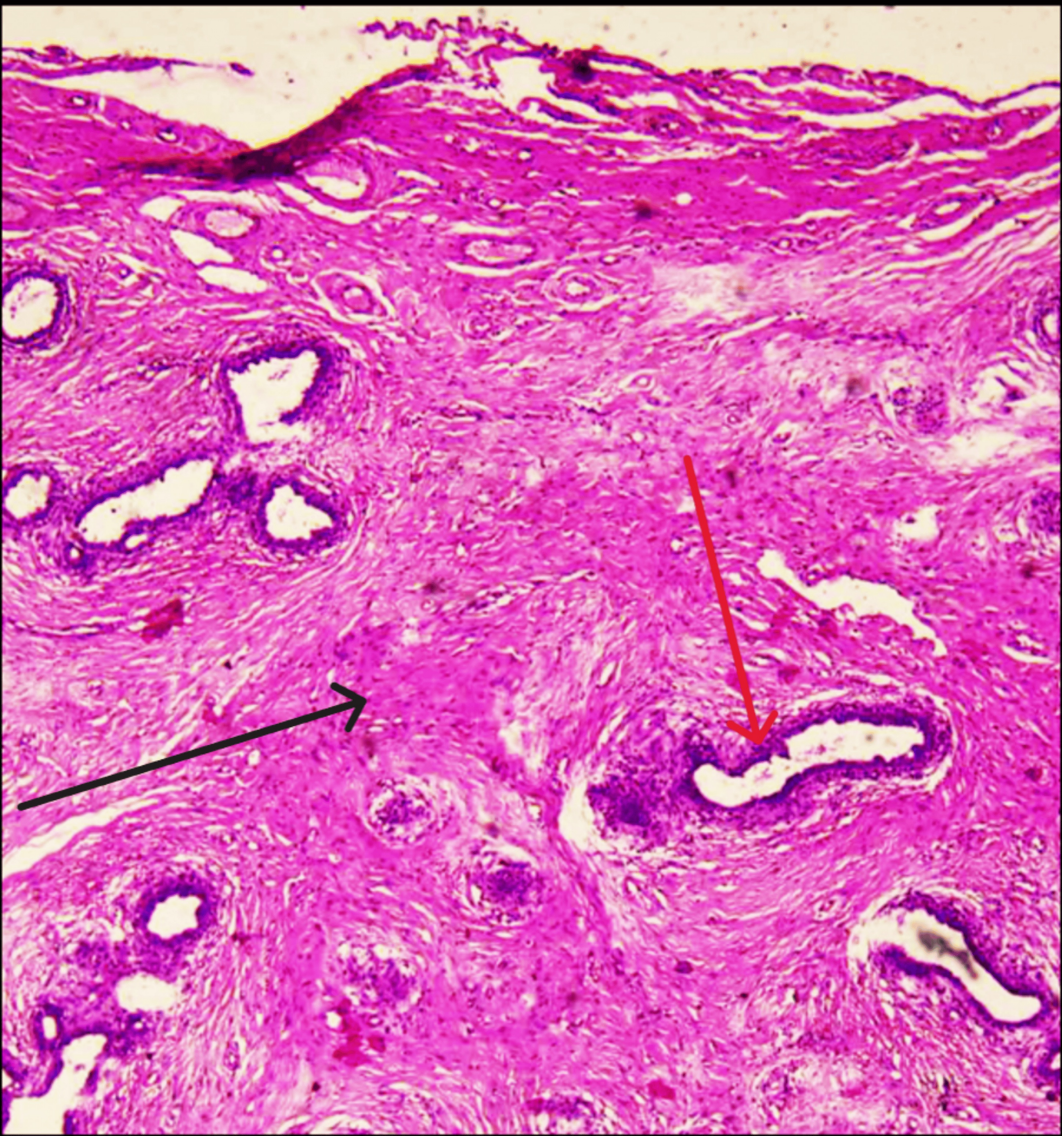
A well-circumscribed biphasic tumor The image shows both glandular (red arrow) and stromal (black arrow) elements.

**Figure 4:**
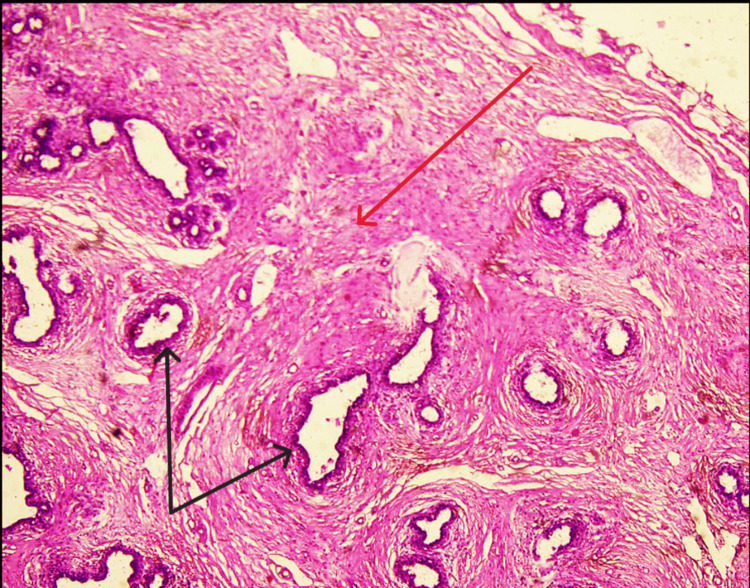
Biphasic tumor with proliferation of both glandular and stromal elements The image shows glandular (black arrows) and stromal (red arrow) proliferation.

**Figure 5:**
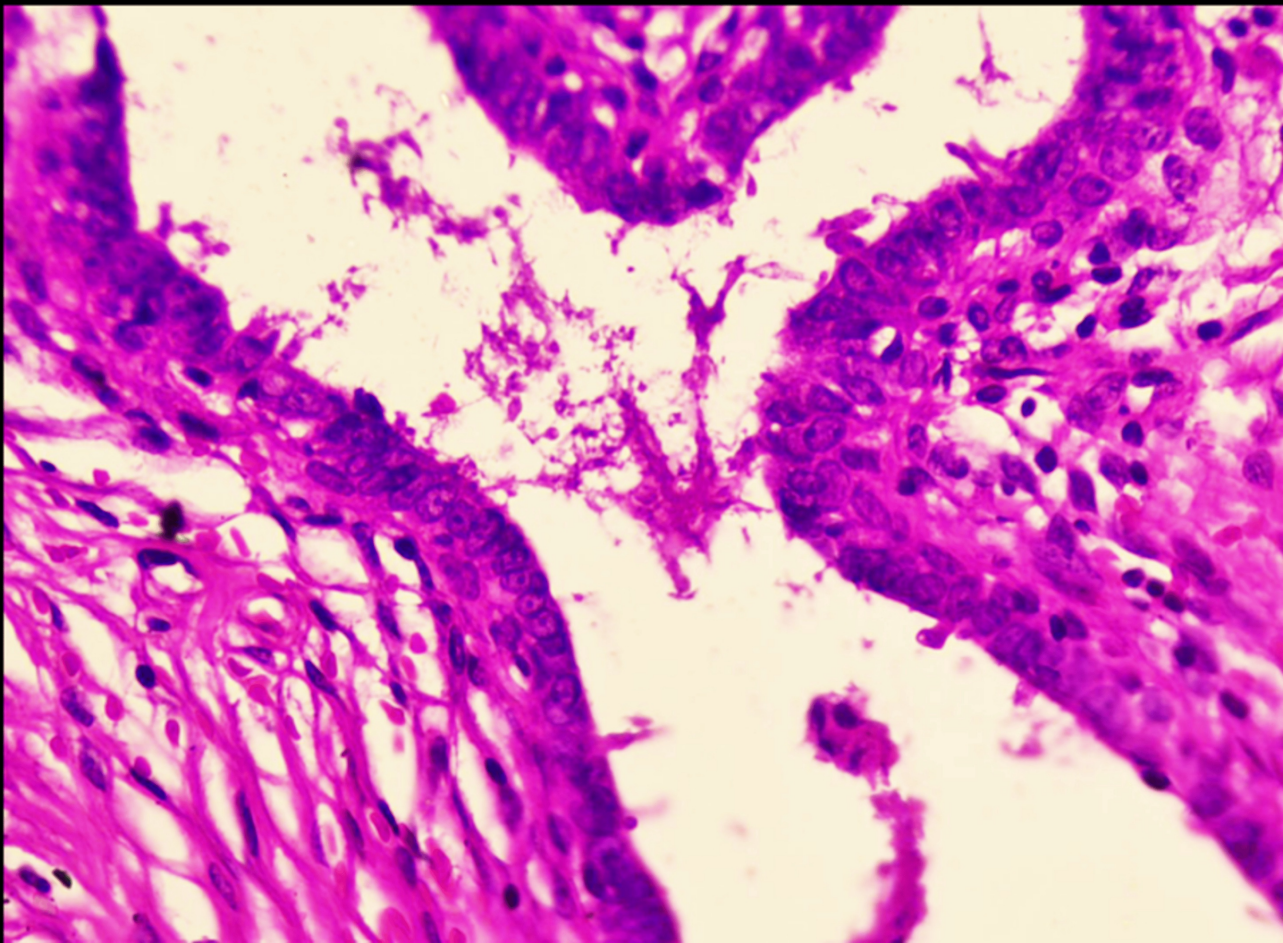
Bilayered epithelium showing inner cuboidal to columnar epithelium and an outer myoepithelium showing cytoplasmic clearing

An initial diagnostic workup and a triple assessment were performed for breasts. Ultrasound of the breast revealed well-circumscribed, round-to-ovoid structures with uniform hypoechogenicity, which was suggestive of a Breast Imaging Reporting And Data System (BI-RADS) 3 lesion with the possibility of a fibroadenoma and was in turn supported by the core needle biopsy (CNB) findings, which reported the same. The USG of the left vulvar and inguinal region was performed, which showed an ovoid lump with heterogeneous echotexture along with CNB of the vulvar lump, which revealed the possibility of EBT. The patient was advised to have an excision biopsy of the vulvar lump (Figure 2), and histopathology reports confirmed the presence of a fibroadenoma in ectopic breast tissue (Figures 3-5). The postoperative period was uneventful. The patient underwent suture removal on postoperative day 10 with no signs of surgical site infection. The patient's follow-up was uneventful as well, with no fresh complaints after the surgery. 

## Discussion

Breast tissue (glandular component/nipple-areolar complex (NAC)/both) occurring at any site other than the pectoral region is termed EBT. During the embryological phase of development, ectodermal ridges are formed on the ventral surface bilaterally, which in turn undergo involution except at the pectoral area, which forms the normal mammary structure. The persistence of the ridges at any location other than the pectoral area results in the formation of EBT. This embryological line is referred to as the mammary line or the ‘milk’ line [5]. The first such case of EBT was described by Hartung in 1872 when they reported a fully formed mammary gland in the vulva [6]. The most common site for EBT, however, is the axilla, accounting for 60%-70% of the cases [7]. The overall incidence of EBT is around 2%-6% in females and 1%-3% in males, with variations among different races, most commonly found in Japanese women. Primary breast cancer in EBT is reported in 0.3%-0.6% of all breast cancers [8]. Kajava classified EBT into eight classes as shown in Table 1 [9,10].

**Table 1:** Kajava classification of ectopic breast tissue Source: [10]

Class	Particulars
Class I	Complete breast with nipple, areola, and breast tissue
Class II	Nipple and glandular tissue with no areola
Class III	Areola and glandular tissue with no nipple
Class IV	Only glandular tissue
Class V	Only nipple and areola but no glandular tissue
Class VI	Only nipple (polythelia)
Class VII	Only areola (polythelia areolaris)
Class VIII	Only a patch of hair (polythelia pilosa)

In some cases, EBT has also been found in sites away from the embryological milk line, and this has been attributed to an aberrant displacement or migratory arrest [11] of the breast primordium or arising from modified apocrine sweat glands in the body [12]. Patients with EBT have also been found to have associated genitourinary abnormalities like polycystic kidney disease, duplication of the ureter, and hydronephrosis, which is explained by the coexisting development of the genitourinary system and the mammary structure [13]. Our patient did not have any congenital coexisting clinical or radiological features.

As mentioned above, EBT can undergo similar changes to normal breast tissue and can cause pain and discomfort due to the proliferation of glandular tissue following hormonal stimulation. A systematic review of 126 cases of vulvar EBT showed benign changes in 57.9% and malignancy in 42.06% of cases [14]. In our case, the patient presented after the vulvar lump that can be classified as Kajava Class IV EBT (as the nipple and areola were absent) started growing in size and became painful. Core needle biopsy and excisional biopsy were helpful in a quick diagnosis that helped in ruling out other causes, especially malignancy.

## Conclusions

This case illustrates the diagnostic challenges posed by the atypical presentation of EBT in the vulva and highlights the importance of considering it in differential diagnoses for vulval masses. Given the potential for malignant transformation, timely diagnosis and management are critical. This report adds to the few documented cases of fibroadenoma in EBT of the vulva and increases awareness for clinicians not to miss or delay such a rare condition.

## References

[1] Giordano SH, Cohen DS, Buzdar AU, Perkins G, Hortobagyi GN (2004). Breast carcinoma in men: a population-based study. Cancer.

[2] Kao GF (1999). Ectopic breast tissue presenting as a mass in the axilla: a case report and review of the literature. Arch Pathol Lab Med.

[3] Sinhasan SP, Pradhan D, Singh P, Kumar A, Kumar A, Kumar A (2016). Ectopic breast tissue: report of a rare case with literature review. J Clin Diagn Res.

[4] Hsueh EC, Hansen SL, Beer TM (2003). Ectopic mammary tissue presenting as an adrenal mass. Ann Surg Oncol.

[5] Shin SJ, Sheikh FS, Allenby PA, Rosen PP (2001). Invasive secretory (juvenile) carcinoma arising in ectopic breast tissue of the axilla. Arch Pathol Lab Med.

[6] Deaver JB, McFarland J, Herman L (1917). The Breast: Its Anomalies, Its Diseases and Their Treatment. Philadelphia, PA: P Blakiston’s Sons & Co.

[7] Sawa M, Kawai N, Sato M, Takeuchi T, Tamaki T, Oura S (2010). Fibroadenoma of the axillary accessory breast: diagnostic value of dynamic magnetic resonance imaging. Jpn J Radiol.

[8] Patel PP, Ibrahim AM, Zhang J, Nguyen JT, Lin SJ, Lee BT (2012). Accessory breast tissue. Eplasty.

[9] Shreshtha S (2016). Supernumerary breast on the back: a case report. Indian J Surg.

[10] Kajava Y (1915). The proportions of supernumerary nipples in the Finnish population. Duodecim.

[11] (2024). Breast embryology: overview, the integument, the embryologic breast. http://emedicine.medscape.com/article/1275146-overview.

[12] Craigmyle MBL (1984). The Apocrine Glands and the Breast. Chichester: Wiley.

[13] Amaranathan A, Balaguruswamy K, Bhat RV, Bora MK (2013). An ectopic breast tissue presenting with fibroadenoma in axilla. Case Rep Surg.

[14] Buitrago-Flechas SM, Barrera-Latorre SJ, Morante-Caicedo C (2021). Ectopic mammary tissue in vulva: case report and systematic literature review. Rev Colomb Obstet Ginecol.

